# Genome analyses of a placozoan rickettsial endosymbiont show a combination of mutualistic and parasitic traits

**DOI:** 10.1038/s41598-019-54037-w

**Published:** 2019-11-26

**Authors:** Kai Kamm, Hans-Jürgen Osigus, Peter F. Stadler, Rob DeSalle, Bernd Schierwater

**Affiliations:** 1 University of Veterinary Medicine Hannover, Foundation, Institute of Animal Ecology, Bünteweg 17d, D-30559 Hannover, Germany; 2 Bioinformatics Group, Department of Computer Science, and Interdisciplinary Center for Bioinformatics, University of Leipzig, Härtelstraße 16-18, D-04107 Leipzig, Germany; 3 Sackler Institute for Comparative Genomics and Division of Invertebrate Zoology, American Museum of Natural History, New York, New York, USA

**Keywords:** Evolutionary ecology, Coevolution, Genome evolution, Bacterial genes, Zoology

## Abstract

Symbiotic relationships between eukaryotic hosts and bacteria range from parasitism to mutualism and may deeply influence both partners’ fitness. The presence of intracellular bacteria in the metazoan phylum Placozoa has been reported several times, but without any knowledge about the nature of this relationship and possible implications for the placozoan holobiont. This information may be of crucial significance since little is known about placozoan ecology and how different species adapt to different environmental conditions, despite being almost invariable at the morphological level. We here report on the novel genome of the rickettsial endosymbiont of *Trichoplax* sp. H2 (strain “Panama”). The combination of eliminated and retained metabolic pathways of the bacterium indicates a potential for a mutualistic as well as for a parasitic relationship, whose outcome could depend on the environmental context. In particular we show that the endosymbiont is dependent on the host for growth and reproduction and that the latter could benefit from a supply with essential amino acids and important cofactors. These findings call for further studies to clarify the actual benefit for the placozoan host and to investigate a possible role of the endosymbiont for ecological separation between placozoan species.

## Introduction

Since the discovery of the metazoan phylum Placozoa^1^ more than a century has passed until it has been recognized to consist of many more species aside *Trichoplax adhaerens*^2–8^ and a first representative genome has been sequenced^9^. Nevertheless, despite recently published additional placozoan genomes^5–7^, the phylum remains poorly understood, including its position in the metazoan Tree of Life (cf.^10^). Placozoans exhibit the simplest morphology of all free-living animals (consisting of only few somatic cell types)^11–14^, which furthermore is almost invariable among different lineages despite the large genetic distances (but see Guidi *et al*.^15^ and Osigus *et al*.^8^). We also know very little about the physiology (e.g.^16–18^) and ecology (e.g.^4,19–21^) of these millimeter sized benthic crawlers (for reviews see^22,23^).

So far, placozoans have been found in temperate to tropical coastal waters up to a depth of 20 meters where they feed on the biofilm of hard substrates. Accordingly, they are less abundant in abrasive environments such as sandy beaches or in areas with high turbidity^4,19,22,24^. Despite being almost indistinguishable at the morphological level, the different genetic lineages (“species”) show substantial differences in their geographic distribution and ecological niches. While some lineages seem restricted to coral reef or mangrove habitats, or may even be endemic to certain locations, others show a remarkable broad latitudinal range^2–4,24–28^. When cultured in the lab, different placozoan lineages differ substantially in their demands for diet and water quality (personal observation, Osigus *et al*.^8^), reflecting the existence of several cosmopolitan (euryoecious) and geographically very restricted (stenoecious or even endemic) species in this phylum^3,4,20^.

Because adaptation to different environmental conditions is not reflected by placozoan morphological variation, ecological separation could be mainly based on physiological adaptations founded in their different genetics. Other contributing factors, however, might include different reproductive strategies or biotic interactions, for example symbiosis with bacteria. Symbiosis, in the broader sense, ranges from parasitism to mutualism (e.g.^29^) and may be a substantial factor contributing to ecological adaptation, by influencing the fitness of the host in general or by shifting its tolerable range of certain environmental variables. Four decades ago, putative endosymbiotic bacteria have been identified in Placozoa by ultrastructural analyses of *Trichoplax adhaerens*^11^. These bacteria seem restricted to a subpopulation of the contractile fiber cells, that are located between the two epithelia, and to developing oocytes^11,30–32^, indicating that they are propagated to the next generation by vegetative as well as sexual reproduction. Some years after the release of the *Trichoplax* reference genome^9^, Driscoll *et al*.^33^ identified several fragments of a bacterial genome in the assembly. Due to the lower coverage of Sanger Shotgun Sequencing (compared to Next Generation Sequencing) used for the *Trichoplax* genome project and the apparent under-representation of bacterial DNA in library preparation, only a fraction of the genome was present (the authors estimated around 20%), which nevertheless allowed the identification of the bacterium as a member of the Midichloriaceae within Rickettsiales^33^.

Rickettsiales form an order of the α-Proteobacteria. They are obligate intracellular bacteria with reduced genome size (typically around 1–1.5 megabases) and gene content which mirrors their incapability to grow and divide outside the host^34,35^. Rickettsiales have evolved a highly diverse set of strategies to manipulate their hosts, ranging from the simple exploitation of the host’s metabolism, to the inference with crucial host pathways such as apoptosis or those involved in reproduction^35^. Although members of the Rickettsiales are mainly known as parasites of mammals and insects, their relationship with eukaryotic hosts also includes mutualism^35^, which requires that both partners benefit, for example by complementing each other’s nutritional demands. This is often the result of a coevolution between the host and its bacterial symbiont, with both partners eventually gaining a net fitness benefit. However, this relationship may be context dependent and evolutionary labile if one of the partners is not strictly dependent on it^29^.

The highly incomplete gene-set present on the genome fragments of the intracellular rickettsial bacterium in *Trichoplax adhaerens* did not allow an assessment of the nature of their symbiosis, which might be an important aspect for understanding placozoan biology^33^. We recently sequenced the genome of the placozoan mitochondrial 16S haplotype H2, *Trichoplax* sp. H2 (strain “Panama”)^5^, which is the most abundant placozoan haplotype around the world and also the most robust lineage for culturing^2–4,28^. The genome sequencing also yielded the novel genome of a single rickettsial endosymbiont which is reported here. We investigated the bacterial genome for important metabolic pathways revealing the principle nature of the placozoan holobiont.

## Results and Discussion

### The placozoan endosymbiont possesses a “typical” compact rickettsial genome that is only distantly related to known genomes of the order Rickettsiales

The genome assembly resulted in 19 scaffolds with a total size of 1.47 Mb. Gene prediction yielded approximately 1,500 genes and a coding density of 87.4% (see Supplementary Table S1 for a summary). With a mean GC content of 27.6% the bacterial genome can be well discriminated from the host genome (32.7% GC^5^). While two of the smaller scaffolds (scaffolds 15 & 17) showed an exceptionally high read coverage (up to 30-fold the average), but are not circular plasmids, the remaining scaffolds showed a roughly 5-fold lower coverage than the host genome (Kamm *et al*.^5^, Supplementary Table S1). This matches the observations of Grell and Benwitz 1971^11^, that the bacteria occur in moderate numbers in only a subpopulation of cells.

Genome completeness was assessed by benchmarking the presence of single-copy orthologs using the BUSCO pipeline^36^ (with the Proteobacteria dataset) and the checkM pipeline^37^ which also infers the appropriate phylogenetic lineage of the benchmarking dataset and thus allows a more accurate estimate. BUSCO yielded 86% complete BUSCOs (3.6% fragmented, 0% duplicated) which is a higher value than is obtained for Candidatus *Midichloria mitochondrii* (78,3%) or a range of complete *Wolbachia* genomes (≤83.8%)^38^. Benchmarking with checkM estimated a completeness of 100% and a possible contamination of 0%. Together, these values are highly indicative that a near complete genome without contamination was rescued from the sequencing of the host genome. Furthermore, several investigated metabolic pathways of the endosymbiont are complete (see below) and many of the genes contributing to a particular pathway are not clustered but are distributed across the genome. The finding of several complete pathways would thus be highly unlikely if more than a minor fraction of the genome was missing. Nevertheless, we thus can only infer a non-functional pathway if multiple evidence is present (i.e. multiple genes are missing or in comparison with the gene complement of related species).

**Figure 1:**
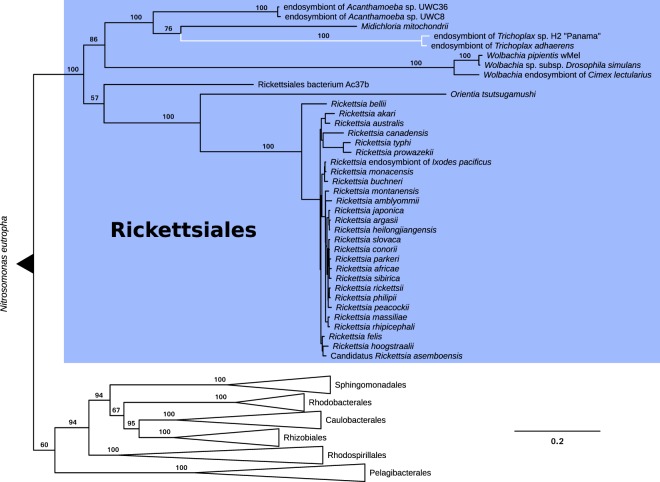
Maximum likelihood phylogenetic analysis of 76 α-Proteobacteria and the two placozoan endosymbionts. The tree is based on a concatenated amino acid alignment comprising the marker genes atpA, atpB, lepA, rplC, rplN, rpoB, rpsE and rpsK. The endosymbionts of *Trichoplax* sp. H2 and *Trichoplax adhaerens* clearly group within the order Rickettsiales and are sister to the Midichloriacea, though separated by long branches from the latter. The tree has been rooted on *Nitrosomonas eutropha*. Branch labels show bootstrap support. Bootstrap values have been omitted if lower than 50 and in the genus *Rickettsia* for clarity. See also Supplementary Fig. S1 for the tree with un-collapsed branches.

Phylogenetic analyses (Fig. 1; Supplementary Fig. S1), that also included homologous genes from the *Trichoplax adhaerens* endosymbiont, confirmed previous analyses that the placozoan endosymbiont belongs to the Rickettsiales; with *Midichloria mitochondrii* (Midichloriaceae)^39^ and the endosymbionts of *Acanthamobae* UWC8/UWC36 (Holosporaceae)^40^ being the closest, but comparatively distant, known relatives with sequenced genomes and proteomes available. The analyses also show that the investigated clonal lineages of *Trichoplax* sp. H2 and *T*. *adhaerens* host closely related but clearly distinguishable rickettsial endosymbiont lineages. While it is possible that the grouping of Midichloriacea, Holosporacea and the two placozoan rickettsial endosymbionts could be due to long-branch attraction, the relationship to Midichloriacea could be further supported by mapping the predicted proteins to orthologous groups using eggNOG-mapper^41^ and retrieving fine grained orthologs (phylogenetically refined orthology assignments). By far the most retrieved fine-grained orthologs belong to *Midichloria* (368, Supplementary Fig. S2), followed by the combined hits from the genus *Rickettsia* (≈120). Another commonality between Midichloriacea, Holosporacea and the placozoan rickettsial endosymbiont is the presence of flagellar genes, as in most free-living α-Proteobacteria, while other Rickettsiales have lost these genes due to their obligate intracellular lifestyle (e.g.^42,43^). However, loss of flagella has happened independently in several lineages of the Rickettsiales and in other, unrelated, bacterial endosymbionts^42,43^ and is thus a weak indicator for relatedness.

Because *Midichloria* and UWC8 were found to be the two most closely related species with sequenced genomes available^40,44^, synteny analyses between the latter and the *Trichoplax* sp. H2 endosymbiont were conducted. Due to the large phylogenetic distances between the taxa we used relaxed parameters, requiring 4 collinear gene pairs and a maximum of 30 intervening genes to define a syntenic block. This approach revealed 58 and 57 regions of synteny between the H2 endosymbiont and *Midichloria* and UWC8, respectively (Supplementary Figs. S3 and S4). Strict collinearity over a long sequence of genes could only be found in a cluster mostly comprised of ribosomal genes (21 and 27 collinear pairs, respectively) or in shorter clusters like the genes for ATPase subunits. This large amount of genomic rearrangement is in concordance with the large phylogenetic distances between these taxa that can be seen in the ML tree.

Since we were not able to close the circular genome, the synteny analyses can also be used to evaluate which of the scaffolds belong to the single chromosome and which are likely plasmids. By applying a threshold of at least 10 gene matches between a scaffold of the endosymbiont and one of the other two Rickettsiales genomes, we conclude that scaffolds 1–8 very likely belong to the endosymbiont chromosome. In addition, scaffold 9 must also belong to the chromosome because it harbors the gene for nuoG of the NADH dehydrogenase complex and three of the genes involved in pantothenate and CoA biosynthesis (see below). The endosymbiont genome then amounts to 1.38 megabases which is a typical size for rickettsial genomes (1–1.5 megabases^34,35^). Nevertheless, it cannot be excluded that the remaining scaffolds are also part of the chromosome.

### The combination of eliminated and retained metabolic pathway components of the endosymbiont indicates a potential for mutualism as well as for parasitism

Because the host provides a relatively stable environment with nutrients, many genes of metabolic pathways needed by free living bacteria have been eliminated from Rickettsiales genomes or even partially transferred to the host genome^34,35^. The compact genome and the total number of predicted genes indicates that this is also true for the endosymbiont found in *Trichoplax* sp. H2. The question remains if the host has any benefit from the bacterium. To investigate this, we applied KEGG pathway mapping^45^ for the predicted genes and the results (Supplementary Tables S2 and S3) support the hypothesis that the endosymbiont provides a selective advantage for the host, although a definite benefit finally has to be proven experimentally.

Like UWC8, and in contrast to many other Rickettsiales and *Midichloria*, the H2 endosymbiont possesses the non-oxidative part of the pentose phosphate pathway for interconversion of sugars. Like *Midichloria* and UWC8, but unlike rickettsias, the genome also contains most enzymes of the canonical glycolysis pathway, but lacks a gene for pyruvate kinase. Instead the bacterium may utilize ppdK (pyruvate, phosphate dikinase) which can boost ATP yields compared to the pyruvate kinase reaction^46^. A complete citrate cycle enables the bacterium to oxidate Acetyl-CoA derived from pyruvate, as is the case in *Midichloria*, UWC8 and rickettsias.

The placozoan endosymbiont possesses almost the same gene complement for oxydative phosphorylation as *Midichloria*^44,45^ but seems to lack clear orthologs of atpF and atpC. However, the genes of the F-type ATPase complex are located in two distinct clusters in the genome, one for the F0 units and one for the F1 units. Downstream of cluster F0 (atpB/E) we found another F0 subunit containing Pfam entry PF02326 that belongs to the same clan as atpF. Downstream of cluster F1 (atpH/A/G/D) another F1 subunit contains the ATP synthase Delta/Epsilon chain of atpC, suggesting that the endosymbiont is capable to form a functional complex V. It is also noteworthy that the endosymbiont genome encodes all three subunits of a cbb_3_-type cytochrome oxidase in the same chromosomal organization like *Midichloria*^44^. This type of cytochrome oxidase is absent in rickettsias and possibly enables oxidative phosphorylation even under low oxygen conditions. In the context of ATP production and consumption it is worth considering whether host and symbiont are capable of exchanging ATP/ADP. We found that the H2 endosymbiont genome encodes the ATP/ADP carrier protein tlcA which can provide the symbiont with host ATP in exchange for ADP. This is an important component of rickettsial energy parasitism^35^ but may also function in reverse such that it equilibrates host and symbiont ATP/ADP across the bacterial membrane^47^.

**Figure 2:**
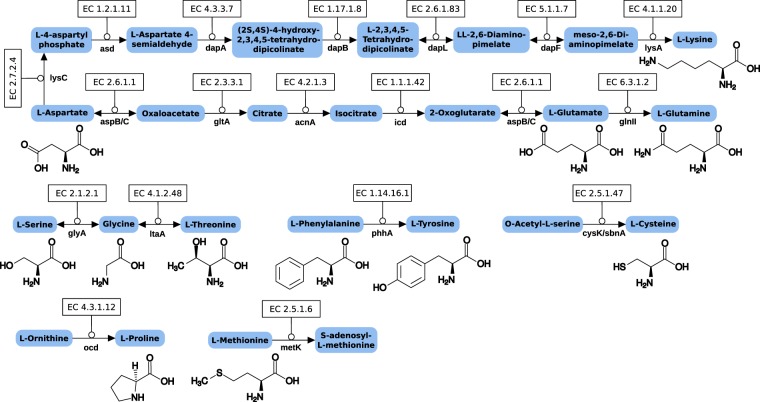
Amino acid synthesis pathways present in the endosymbiont of *Trichoplax* sp. H2. The symbiont’s genome has retained only few pathway genes for de-novo synthesis from metabolic intermediates. Most remarkable is the retained capability to synthesize the essential amino acids lysine and threonine.

The capabilities of the endosymbiont to synthesize amino acids (Fig. 2) show a mixture of traits that are parasitic and traits that could be beneficial for the host. On the one hand it has lost the pathways for de-novo synthesis of most amino acids from metabolic intermediates and is thus dependent on the host cytoplasm for their supply. However, starting from oxaloacetate (citrate cycle) the endosymbiont is able to synthesize aspartate, glutamine, glutamate and, most important, the essential amino acid lysine. Surprisingly, the *Trichoplax* sp. H2 nuclear genome^5^ also harbors two genes (dapF, lysA) of bacterial origin which catalyze the two last steps in lysine synthesis and could possibly contribute to the pathway. The symbiont is further able to convert phenylalanine into tyrosine, as well as serine, glycine and the essential amino acid threonine into one another. The capacity to synthesize lysine and to convert glycine into threonine is also the most important deviation from *Midichloria*, which has a similar reduced gene set but lacks the enzymes dapB, dapF and ltaA^45^.

**Figure 3:**
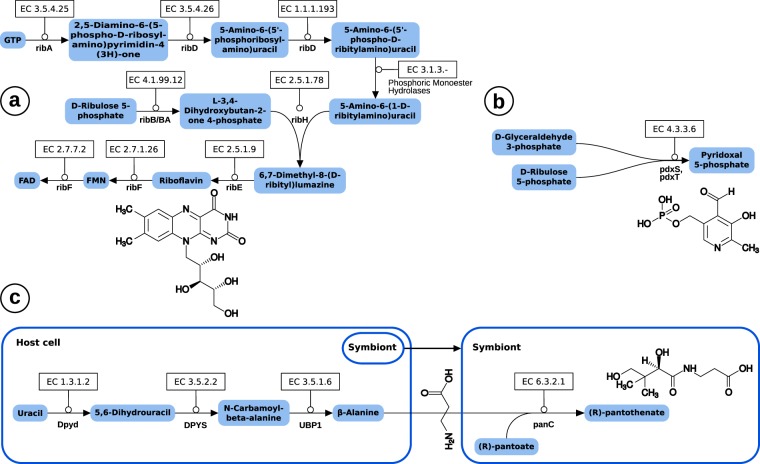
Important cofactor biosynthesis pathways of the endosymbiont. The symbiont is able to provide the cofactors **(a)** riboflavin and (**b**) pyridoxal 5-phosphate (vitamin B6) for the host. **(c)** The symbiont possesses panC which enables pantothenate synthesis from β-alanine and pantoate but has lost panD for β-alanine synthesis. The latter can be provided by the host via degradation from uracil.

The endosymbiont might also provide a crucial supply of cofactors needed in metabolic pathways (Fig. 3). For example, its genome encodes all enzymes necessary to synthesize riboflavin from ribulose-5-phosphate and GTP. These enzymes are absent in rickettsias and most of them have been lost in *Midichloria*, while the endosymbiont of *Acanthamoeba* UWC8 has retained a functional pathway for riboflavin synthesis^45^. In insect endosymbionts of the genus *Wolbachia*, the genes for riboflavin synthesis appear to be conserved among several species and it has been shown that riboflavin provisioning significantly contributes to host fitness^48^. The retention of this pathway in the H2 “Panama” endosymbiont, in contrast to many other Rickettsiales, likewise indicates that the placozoan host is not able to sufficiently provide flavines through his diet and is in need of flavines from other sources.

Using the enzymes pdxS and pdxT the placozoan endosymbiont is able to build pyridoxal phosphate. Another benefit for the host is probably the symbiont’s ability to build pantothenate (and CoA) from β-alanine and pantoate. Interestingly, the bacterium has lost the capacity to synthesize β-alanine itself from l-aspartate because it has lost panD. Exactly the same phenomenon can be observed in several obligate insect endosymbionts belonging to the unrelated γ-Proteobacteria^49^. As in the latter holobionts, *Trichoplax* sp. H2 is capable of complementing the missing panD, and thus β-alanine supply, via a three-step uracil degradation pathway. Other examples of cofactor biosynthesis pathways present in the H2 endosymbiont include almost complete pathways for biotin and protoheme synthesis. In the latter two cases it is remarkable that only one enzyme each of the complex pathways for biotin (bioH) and protoheme (hemG) is missing and that the same gene set can be found in *Midichloria*. While the missing hemG can be complemented by the host (PPOX)^5^, this is not the case for bioH and the question remains why the other genes have been retained although many of them only participate in biotin synthesis.

### The composition of the flagellar assembly pathway suggests the absence of a motile phase to switch between different hosts

**Figure 4:**
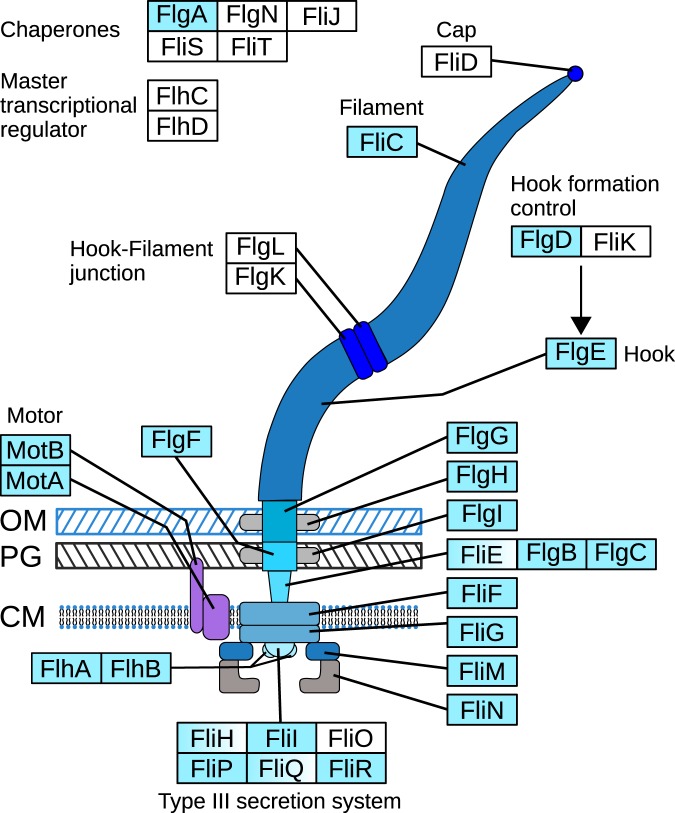
Flagellar assembly pathway components present in the H2 endosymbiont based on KEGG-mapping. The presence of a gene is indicated by filled boxes. Half-filled boxes indicate genes that were not identified by KEGG but harbor the necessary domains. Most genes needed for a functional flagellum are present in the genome but the genes for the hook-filament junction and the filament cap gene fliD are absent, suggesting that the bacterium is not able to build a functional flagellum. Because most genes of the type III secretion system and the basal body are present, the rudimentary flagellum might still function as a substrate export system. OM = outer membrane, PG = peptidoglycan layer, CM = cytoplasm membrane (simplified after Toft & Fares 2008^42^ and KEGG pathway maps).

A further pathway which may become obsolete in a tight host-symbiont relationship is the flagellar assembly pathway. The primary function of the flagellum is motility and the expression and synthesis of more than 30 proteins^50^ requires a significant amount of energy from the bacterium. The obligate intracellular rickettsias are generally described as non-flagellated^43^ and different grades of flagellar gene losses have been observed in other bacterial endosymbionts^42^. As mentioned above, we found several flagellar genes in the H2 endosymbiont: 21 genes of the assembly pathway could be assigned by KEGG mapping (Fig. 4). In addition, we found weak fliH, fliQ and fliE orthologs with recognizable domains. This is at least 2 genes fewer compared to the KEGG flagellar pathway map of *Midichloria*. The missing genes include flgL and flgK which are required as a junction between the hook and the filament, as well as the filament cap fliD, which enables proper assembly and folding of the filament subunits^50^. The master transcriptional regulators and almost all chaperones of the assembly pathway are also missing. This suggests that the H2 endosymbiont is not able to build a functional flagellum, indicating that it does not have a motile phase for switching between different generations or hosts.

For the *Buchnera* endosymbionts of aphids it has been proposed that differential gene loss of flagellar genes among the different species may be governed by a tight metabolic and biochemical interaction between host and symbiont and that the retained genes possibly mediate protein export to the host^42^. The presence of seven out of eight genes of the flagellar export apparatus and all basal body genes in the placozoan endosymbiont suggests that the same transition in function has happened here.

### Possible adaptations to modulate host defense

In sum we found evidence for an intimate association between the placozoan host and the bacterial symbiont which suggests that the latter is not able to thrive on its own. The host may even complement some of the fragmented endosymbiont pathways. This points to a coevolutionary history and raises the question how this relationship has been established, mediated and maintained. For the secretion of putative host modulating effector molecules, the endosymbiont genome encodes a typical Rickettsiales vir homolog (*rvh*) Type IV secretion system (T4SS)^51^. Like many other Rickettsiales^51^, it only lacks virB1, virB5 and virB7 but includes the typical four homologs of virB6, two of virB4 and two of virB8 (Supplementary Tables S2 and S3). On the other hand, its general secretion system (sec-SRP) differs from other Rickettsiales^52^, including *Midichloria*^45^, in the lack of the subunits secE and secG of the protein translocation channel SecYEG, suggesting that the system is not functional. From the side of the effectors, it has been suggested that ankyrin repeat (ANK) containing proteins, secreted by endosymbionts, play an important role in ensuring their survival in the host, because they have been shown to alter host gene expression and are significantly enriched in the genomes of bacterial symbionts^53^. However, we found only 6 predicted genes containing ANK-repeats in the genome of the H2 endosymbiont; as opposed to, for example, 58 in *Wolbachia* sp. wPip Pel or 28 in *Rickettsia belii*^53^, indicating that expansion of ANK-proteins in the H2 endosymbiont was not favored by selection.

On the other hand, the endosymbiont appears to use a rather evasive strategy to ensure its survival in the host. We found that selection might have strongly acted upon the CpG-content of the symbiont’s genome. Compared to related endosymbiotic α-Proteobacteria like *Wolbachia*, *Midichloria* or rickettsias, the H2 endosymbiont has a roughly three times lower occurrence of CpG-motifs. The γ-Proteobacterium *Escherichia coli* even exhibits an eleven times higher frequency (Table 1). This low CpG-amount is comparable to that of the unrelated pathogenic bacterium *Clostridium perfringens* whose DNA has been shown to elicit the weakest immuno-stimulatory response in mammalian cell cultures, compared to other bacteria^54^.

**Table 1:** Examples of bacterial genomes and their CpG-motif frequency.

Genome	Class	%GC	CpG motifs/kb (single strand)	Accession
*Escherichia coli*	γ-Proteobacteria	51	76.5	NC_020518.1
Candidatus *Midichloria mitochondrii* IricVA	α-Proteobacteria	37	23.8	NC_015722.1
*Rickettsia bellii*	α-Proteobacteria	32	20.4	NC_007940.1
*Wolbachia* endosymbiont of *Drosophila melanogaster*	α-Proteobacteria	35	20.2	NC_002978.6
H2 endosymbiont	α-Proteobacteria	28	7.1	NPHZ00000000
*Clostridium perfringens*	Clostridia	29	5.2	NC_003366.1

Among the α-Proteobacteria, the *Trichoplax* sp. H2 endosymbiont is characterized by an extremely low CpG-content.

The endosymbiont’s extremely low CpG content indicates a selective pressure exerted by the host’s ability to detect CpG-DNA or other pathogen associated molecular patterns (PAMPs) - presumably by pattern recognition receptors (PRRs) of an innate immune system which is the main defense against invading pathogens in invertebrates^55,56^. However, apart from defense, innate immunity also seems to play a fundamental role in managing symbiotic interactions, such that the animal host is able to discern, tolerate and promote beneficial bacteria^57^. Innate immunity has been well known in animals as basal as Cnidaria (e.g.^58^) and, more recently, we were able to identify several components of innate immunity also in Placozoa^59^. It can thus be assumed that *Trichoplax* sp. H2 is able to effectively manage the endosymbiotic bacteria, despite the low genomic CpG content, which is also reflected by their restriction to certain cell populations.

### Possible outcomes and context dependency of the relationship

While most of the described pathways are solely required by the symbiont and some represent parasitic traits, others have the potential to provide a benefit for the host. Collaboration and the complementation of pathways requires the expression of the (complementary) metabolism genes of symbiont and/or host and the presence of dedicated transport mechanisms for exchange of the metabolites^49^. In the absence of endosymbiont gene expression or other functional data we cannot decisively tell whether the bacterium provides an advantage for the host and if some of the fragmented pathways are complemented. However, in the case of fragmented pathways, it has been argued that their evolutionary retention strongly indicates that they are functional through complementation by (e.g.) the host^49,60^. In the case of exporters, we were not able to annotate amino acid ABC transporters but we identified two possible amino acid exporters carrying a LysE type translocator domain, one of which was annotated by KEGG as L-lysine exporter family protein LysE/ArgO (Supplementary Table S2 and S3). This suggests that certain amino acids can be delivered to the host. Regarding cofactor provisioning there is generally little known about the cellular mechanisms that facilitate their export. For example, only two bacterial exporters have been characterized that are involved in flavine secretion^61^. The two characterized exporters belong to the Multi-Antimicrobial Extrusion Protein (MATE) family and we were able to identify one member in the H2 endosymbiont genome, but any prediction of its substrate(s) would be highly speculative as long as clear orthologs in related bacteria have not been functionally characterized. Nevertheless, the benefit of flavines, provided by bacterial symbionts, for insect hosts has been documented without knowledge of the involved transport mechanisms^48^. If the endosymbiont is indeed missing the yet to be identified exporters, it is also possible that exchange of metabolites is enabled by mechanisms that are enforced by the host, e.g. similar to those that take place at the host-derived symbiosomal membrane in insect bacteriocyte cells^62^. Another possible mechanism of the host could be the use of antimicrobial peptides (AMPs) which appear to be widespread in eukaryotes^63^. Besides their function as effectors of innate immunity, AMPs have been proposed to play a major role in the metabolic integration of eukaryotic hosts and bacterial symbionts through membrane permeabilization, especially in the case of endosymbiotic bacteria with strongly reduced genomes^63^.

It is also very likely that any metabolic collaboration between the placozoan host and its symbiont is dependent on the environmental context^64^. Provided with a food source that contains all essential amino acids and cofactors, the relationship would have no benefit for the host and could become entirely parasitic. On the other hand, on less optimal food sources the endosymbiont could be beneficial for the host, provided that it is able to deliver the missing nutrients (cf. Russel *et al*.^65^). To date, we have no data about the diet of placozoans in the wild and even from lab cultures no experimental data are available (also owed to the fact that the composition of the biofilm on which they feed is more or less erratic). However, we can expect that the diet of a given placozoan lineage differs at different times and locations and is not always optimal. The outcome of the relationship could thus vary along a continuum from mutualism to parasitism, depending on the environment.

### Note added during review

During the review process, a report has been published by Gruber-Vodicka *et al*.^66^ who describe a different placozoan Rickettsiales endosymbiont named Candidatus *Grellia incantans*. Its placozoan host has been captured in the Pacific Ocean (Hawaii) and has been assigned to the mitochondrial 16S haplotype H2. The host *Trichoplax* sp. H2 reported here and in the genome publication of Kamm *et al*.^5^ originates from the Caribbean Sea (Panama)^2^. Interestingly, the *Grellia* endosymbiont differs in several aspects from the endosymbiont described here and also from the one in the host *Trichoplax adhaerens* (“RETA1” of the placozoan mitochondrial 16S haplotype H1): The *Trichoplax adhaerens* and the *Trichoplax* sp. H2 “Panama” endosymbionts show only 98% and 98.3% 16S identity to *Grellia incantans*, respectively. In contrast, the first two endosymbionts show 99.3% 16S identity and in the 16S maximum likelihood phylogenetic tree both segregate together in a separate clade from *Grellia incantans* (Supplementary Fig. S5). The amount of divergence in the 16S rDNA between the three bacteria indicates that the rickettsial endosymbionts of *Trichoplax adhaerens* and *Trichoplax* sp. H2 “Panama” are different strains of the same species while *Grellia incantans* represents a different species of the same genus^67^.

The *Grellia* endosymbiont also differs partially from the pathway components identified in the endosymbiont of *Trichoplax* sp. H2 “Panama”. For example, *Grellia* lacks the capability to synthesize the essential amino acid lysine, to convert glycine into threonine and was found to encode more than 30 genes for a fully functional flagellum. Together, these studies further highlight the necessity to investigate and evaluate the variability of the placozoan microbiome in future studies.

At present, we can only speculate about the reasons for the presence of less related endosymbionts in two placozoans of the same mitochondrial 16S haploytpe on the one hand, and two closer related endosymbionts in two placozoans of a different haplotype on the other hand. One plausible explanation would be that placozoans eliminate their endosymbiont(s) under certain conditions and subsequently take up related bacteria, leading to different endosymbionts (or combinations) in different populations. It is also possible that endosymbionts are differentially propagated to the next generation via sexual reproduction (cf. Grell 1972^30^, Eitel *et al*.^32^). If mitochondria and endosymbionts are differentially propagated, new combinations of both may also occur as a result of interbreeding between related placozoan hosts (cf. Kamm *et al*.^5^).

## Conclusions

The genomic analyses of the placozoan endosymbiont indicates that *Trichoplax* sp. H2 “Panama” (as well as its relative *Trichoplax adhaerens)* harbors a rickettsial endosymbiont with reduced genome size and gene number that is dependent on the host for growth and reproduction. On the other hand, several of the endosymbiont’s retained metabolic pathways could be advantageous for the placozoan host. In particular, a supply with essential amino acids and important cofactors by the symbiont could positively affect the placozoan host’s ability to exploit less favorable food sources and thus enable it to colonize a broader range of habitats. In summary, the genetic data support a potential for a mutualistic relationship between the bacterium and *Trichoplax* sp. H2 but also for parasitism. More experimental data are needed to verify and quantify a benefit for the host and how it is dependent on the environmental context. These findings also call for follow-up studies to unravel a possible interplay between endosymbiont composition and ecological separation of species in the phylum Placozoa.

## Methods

### Animal material, genome sequencing and assembly

The placozoan lineage *Trichoplax* sp. H2 “Panama” has been collected in the Caribbean, Bocas del Toro, Panama in 2002^2,24^ and is cultured as a clonal strain in our lab as previously described^3^. Prior to genomic DNA isolation the animals were transferred to a clean glass petri dish, starved for at least two days and washed several times with clean artificial seawater (ASW). Genomic DNA was extracted using a standard phenol-chloroform nucleic acid extraction protocol^68^ with subsequent RNase digest.

Two genomic DNA libraries were constructed for *Trichoplax* sp. H2: One paired-end library with a targeted insert size of 300 bp was prepared following the Illumina protocol “Preparing Samples for Paired-End Sequencing” protocol (Part # 1005063 Rev. A, June 2008). This library was sequenced on an Illumina GAIIx instrument (2 × 72 bp) at Ambry Genetics (California, USA) resulting in 85.5 million paired-end reads. These reads were eventually not used for the *Trichoplax* sp. H2 genome assembly but only for the re-assembly of the endosymbiont genome (see below). The second paired-end library had a targeted insert size of 500 bp with 150 bp read length and was the library used for genome assembly of *Trichoplax* sp. H2 (for details see Kamm *et al*.^5^). The number of clonal animals used for library preparation have been approximately 2,000 for the 72 bp library and 500 for the 150 bp library.

The genome assembly of *Trichoplax* sp. H2 and the subsequent procedure used for identification and removal of the endosymbiont scaffolds has been described in Kamm *et al*.^5^. Because the assembly pipeline used for *Trichoplax* sp. H2 is intended for diploid organisms with a reasonable grade of heterozygosity, the endosymbiont genome was re-assembled with Spades v3.8.1^69^ using both paired-end libraries described above to increase the coverage of the endosymbiont genome. To extract the reads belonging to the endosymbiont genome from both libraries, these were iteratively mapped against the scaffolds identified as belonging to the endosymbiont using the Geneious mapper (v8.1^70^). After three iterations no further overlapping reads were detected. All mapping reads were extracted along with their mates and assembled using Spades with default parameters (including BayesHammer correction) and automatic as well as manually set iterations of k-mer size. The best assembly was produced with the k-mer iteration 25,35,55,77,99,103,105. Read coverage was assessed by mapping the paired-end reads against the endosymbiont assembly with BWA MEM^71^ and calling the mapping rate with Samtools 1.2^72^.

### Gene prediction and annotation

Gene-prediction for the endosymbiont (and for better comparison also for *Midichloria mitochondrii* and Endosymbiont of *Acanthamoeba* sp. UWC8) was carried out using the evidence-based Maker annotation pipeline (v2.31.8)^73^ along with prokaryotic GeneMark.hmm (3.36)^74^. Species specific model parameters for GeneMark.hmm were constructed using self-training with the respective genomes in GeneMarkS (4.32)^74^. Evidence given to Maker was a protein set consisting of UniProt entries of Endosymbiont of *Acanthamoeba* sp. UWC8 & UWC36, *Midichloria mitochondrii*, *Rickettsia bellii* (strain RML369-C), *Rickettsia rickettsii* (strain Sheila Smith) and *Wolbachia* endosymbiont of *Drosophila simulans*. Additionally, a prediction for the endosymbiont derived from the prokaryotic GeneMark.hmm online-server, using model parameters of *Midichloria mitochondrii*, was added to the evidence protein file.

Genome completeness was assessed by benchmarking the predicted proteins for the presence of single-copy orthologs with BUSCO (3.1.0^36^) and the Proteobacteria dataset (proteobacteria_odb9). For a comparison, the proteome of *Midichloria* (UniProt ID UP000006639) was also benchmarked with BUSCO. Completeness and potential contamination of the genome was also assessed with checkM (v1.0.18^37^) using our predicted gene set as well as the implemented Prodigal^75^ prediction software. Both gene sets returned identical values. Functional annotation of the predicted proteins was carried out using InterProScan (5.19–58.0)^76^ with the following analyses: CDD-3.14, SignalP_EUK-4.1, PIRSF-3.01, Pfam-29.0, SignalP_GRAM_POSITIVE-4.1, TMHMM-2.0c, PRINTS-42.0, ProSiteProfiles-20.119, PANTHER-10.0, Coils-2.2.1, Hamap-201605.11, ProSitePatterns-20.119, SUPERFAMILY-1.75, ProDom-2006.1, SMART-7.1, SignalP_GRAM_NEGATIVE-4.1, Gene3D-3.5.0 and TIGRFAM-15.0. Annotation of predicted proteins also included BLASTP^77^ searches against SwissProt (cutoff e-value 1e-5) and KEGG pathway mapping using KAAS^78^. To retrieve fine-grained orthologs for the endosymbiont’s predicted proteins, the standalone eggNOG-mapper 0.9.0^79^) was used with the database for α-Proteobacteria (aproNOG). CpG-motif identification for the H2 endosymbiont genome and other representative bacterial genomes was carried out with CPGREPORT of the EMBOSS suite (v6.6.0.0^80^).

### Phylogenetic analyses

To infer the relatedness among the endosymbionts of *Trichoplax adhaerens* and *Trichoplax* sp. H2 and to other α-Proteobacteria, maximum likelihood phylogenetic analyses were conducted using a concatenated amino acid alignment of marker genes. Because the endosymbiont sequences retrieved from the *Trichoplax* reference genome are fragmented, contain several stretches of Ns and represent only a fraction of the genome, the choice of marker genes was limited and for the final set we chose the genes rpoB, rplN, rpsK, rplC, rpsE, atpA, atpB and lepA. In addition to the two endosymbionts, 76 α-Proteobacteria were included and the β-proteobacterium *Nitrosomonas eutropha* as the outgroup. The respective genes from the H2 endosymbiont were taken from the Maker prediction and for the *Trichoplax adhaerens* endosymbiont the genes were predicted from the retrieved scaffolds using the prokaryotic GeneMark.hmm online-server with model parameters of *Midichloria mitochondrii*. The *Trichoplax adhaerens* endosymbiont rpoB gene was found to be interrupted by 400 bp of Ns on scaffold_1380 of the reference assembly and the gap was therefore closed by PCR amplification and Sanger sequencing.

The amino acid sequences were aligned separately for each marker using Muscle^81^, implemented in Unipro UGENE^82^, and inspected, corrected and clipped by eye. The eight alignments were then concatenated, resulting in an alignment with 2733 amino acid positions, and analyzed with RAxML (8.2.0^83^). RAxML was run with the gamma model of rate heterogeneity and automatic protein model assignment using the AICc criterion. Rapid bootstraping was performed using the MRE-based Bootstopping criterion. The tree was visualized in FigTree 1.4.2^84^ and rooted on *Nitrosomonas eutropha*. Accessions for the proteins of the used dataset and details of the PCR amplification of the *Trichoplax adhaerens* endosymbiont rpoB gene are given in Supplementary Table S4.

To infer the relatedness between the recently described Candidatus *Grellia incantans* and the other two placozoan endosymbionts, maximum likelihood phylogenetic analyses were also conducted with the 16S marker gene and a smaller set of taxa from the order Rickettsiales. The 16S gene of *Grellia* has been obtained by personal communication from Gruber-Vodicka *et al*. 2019^66^. A larger fragment of the *Trichoplax adhaerens* endosymbiont 16S gene has been obtained by blasting the 590 bp 16S fragment described in Driscoll *et al*. 2013^33^ against the *Trichoplax adhaerens* transcriptome^5^. The retrieved sequence showed 100% identity and was blasted against the *Trichoplax* sp. H2 endosymbiont genome to identify the corresponding sequence. All other 16S fragments were obtained from GenBank (accessions in Supplementary Table S4). The 16S sequences were aligned with Muscle^81^ implemented in Unipro UGENE^82^. The alignment was then trimmed to equal length and refined using Gblocks (0.91b^85,86^) with default parameters, resulting in an alignment length of 1,431 bp. The best suited substitution model was inferred with jModelTest (2.1.10^87,88^) and phylogenetic analyses were conducted using RAxML (8.2.0^83^) with the substitution matrix GTR and the GAMMA + P-Invar model of rate heterogeneity. Rapid bootstrapping was performed with 1,000 replicates and the tree was visualized in FigTree 1.4.2^84^.

### Synteny analyses

For synteny analyses between the endosymbiont genome and the genomes of *Midichloria mitochondrii* and Endosymbiont of *Acanthamoeba* sp. UWC8 based on gene models generated by Maker, the SynMap pipeline at CoGe (genomevolution.org)^89^ was used, implementing LAST^90^ for finding best protein pairs and DAGchainer^91^ for identification of collinear pairs. Because the endosymbiont genome is not closely related to either *Midichloria mitochondrii* or the Endosymbiont of *Acanthamoeba* sp. UWC8, synteny analyses were conducted with relaxed parameters, requiring 4 collinear pairs with a maximum of 30 intervening genes to call a syntenic region.

### Metabolic pathways of the endosymbiont

Metabolic pathways of the endosymbiont were inferred using the KEGG annotation (see above)^78^. If KEGG mapping did not yield a positive hit for certain genes involved in a particular pathway, the InterProScan 5 annotation was searched for the presence of the respective conserved domains. Still missing flagellar genes were additionally searched for by blasting a set of orthologous proteins against the endosymbiont and the host genome (cutoff 1e-2). The protein set consisted of up to 25 Swiss-Prot entries per gene (depending on the number of deposited genes) and up to 25 TrEMBL entries of the taxon Rickettsiales per gene (α-Proteobacteria if not present).

## Supplementary information

Supplementary Information

Supplementary Table S2

Supplementary Table S3

Supplementary Table S4

## Data Availability

The annotated Whole Genome Shotgun project of the Rickettsiales endosymbiont of *Trichoplax* sp. H2 has been deposited at DDBJ/ENA/GenBank under the accession NPHZ00000000. The version described in this paper is version NPHZ01000000. Individual genes or products described in this paper are indicated by their locus_tag. Genomic Paired-End Illumina reads of *Trichoplax* sp. H2 have been deposited at the NCBI Sequence Read Archive under the accessions SRR5934055 (150 bp reads; see^5^) and SRR5934125 (72 bp reads). The Maker annotated genomes of Endosymbiont of *Acanthamoeba* sp. UWC8 (accession NZ_CP004403) and Candidatus *Midichloria mitochondrii* IricVA (accession NC_015722) have been deposited at the CoGe Comparative Genomics website under the genome IDs 31989 and 31991, respectively. Further datasets supporting this article have been uploaded as part of the supplementary information.
