# Supplementary material for: Genome analyses of a placozoan rickettsial endosymbiont show a combination of mutualistic and parasitic traits
Source: Sci Rep. 2019 Nov 26;9:17561. doi: 10.1038/s41598-019-54037-w (PMC6879607; doi:10.1038/s41598-019-54037-w)
Supplement: Supplementary file 1 — Supplementary Information [file 41598_2019_54037_MOESM1_ESM.pdf]

Genome analyses of a placozoan rickettsial endosymbiont show a combination of mutualistic and parasitic traits

Kai Kamm, Hans-Jürgen Osigus, Peter F. Stadler, Rob DeSalle, Bernd Schierwater

Supplementary Information

Supplementary Table S1

Summary of the endosymbiont's genome assembly and annotation. Note: Read coverage is given separately for the two used libraries for better comparison with the read coverage of the host genome assembly (see Kamm et al. 2018[1]) for which only the 150bp read library was used and which had a read coverage around 80x.

**Assembly & Annotation Statistics**	**Endosymbiont of *Trichoplax* sp. H2**
Assembly Size	1,474kb
Scaffolds	19
Largest Scaffold	398kb
N50	255kb
%GC	27.6
Ns	0.09/kb
Coverage 150bp reads	16.4x
Coverage 72bp reads	6.8x
Predicted protein coding genes	1,473
Coding density	87.4%
Mean CDS size	874.2bp
Mean protein length	290.4AA
Predictions with SwissProt hit	967
Predictions with InterProScan result	1,305
Predictions with EggNOG hits (aproNOG)	1,191
Refined EggNOG hits (aproNOG)	1,162
KEGG KO total/non redundant	760/706

Supplementary Table S2 (separate XLSX).

KEGG mapping results of the endosymbiont's predicted proteins. Accessions of the proteins are given as locus_tags of the annotated genome deposited at DDBJ/ENA/GenBank.

Supplementary Table S3 (separate XLSX).

Annotated genes (including their locus_tags) of the endosymbiont involved in the described metabolic pathways (amino acid synthesis, cofactor synthesis, carbohydrate metabolism, oxidative phosphorylation, flagellar assembly pathway, T4SS).

Supplementary Table S4 (separate XLSX).

Accessions of the proteins and 16S sequences used for phylogenetic analyses. The marker proteins of the *Trichoplax* sp. H2 “Panama” endosymbiont are indicated with their respective locus_tag of the annotated genome deposited at DDBJ/ENA/GenBank. For the marker proteins of the Rickettsiales endosymbiont of *Trichoplax adhaerens,* the underlying genomic scaffolds of the reference genome are listed instead, as well as the primers (and resulting PCR product) used for closing the gap of the rpoB gene.

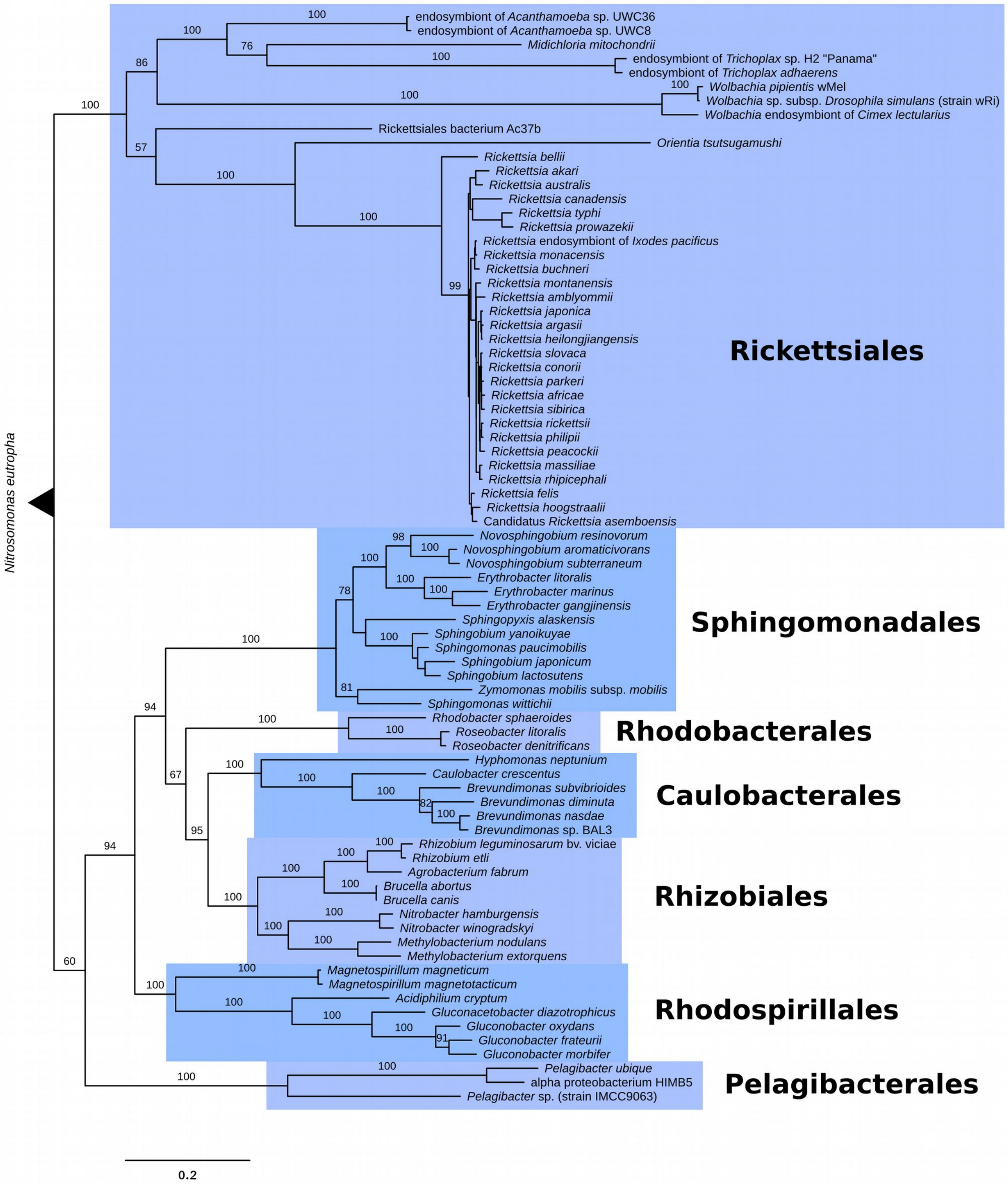

Supplementary Figure S1

Maximum likelihood phylogenetic analysis between 76 α-Proteobacteria and the two placozoan endosymbionts based on a concatenated amino acid alignment. The marker genes used were atpA, atpB, lepA, rplC, rplN, rpoB, rpsE and rpsK. The has been rooted on the β-proteobacterium *Nitrosomonas eutropha*. Branch labels show bootstrap support. Bootstrap values have been omitted if lower than 50 and in the genus *Rickettsia* for clarity.

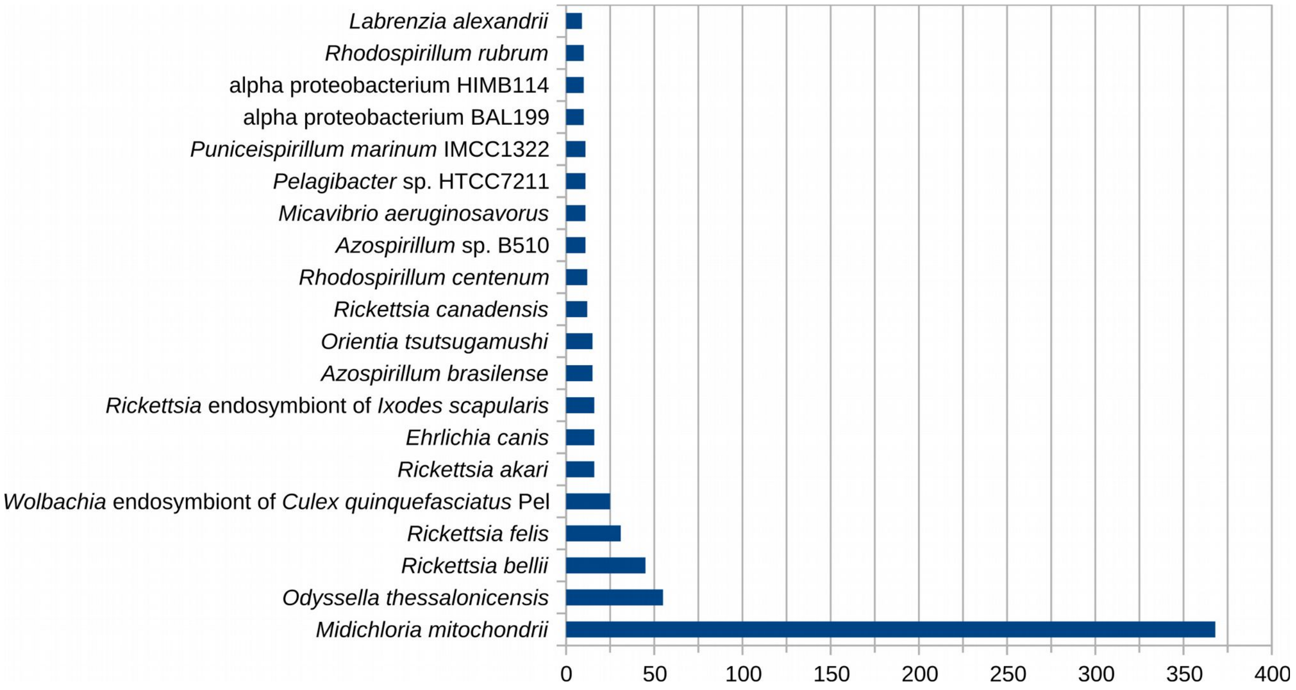

Supplementary Figure S2

Top 20 species of the refined EggNOG hits (fine grained orthologs) from orthologous mapping of the endosymbiont's predicted proteins using the eggNOG mapper with the α-Proteobacteria dataset (aproNOG). Of the 1,473 predicted genes, 1,191 could be assigned to orthologous groups and 1,162 received a fine grained ortholog. Most of the latter belong to *Midichloria mitochondrii*, followed by the combined hits of the genus *Rickettsia.*

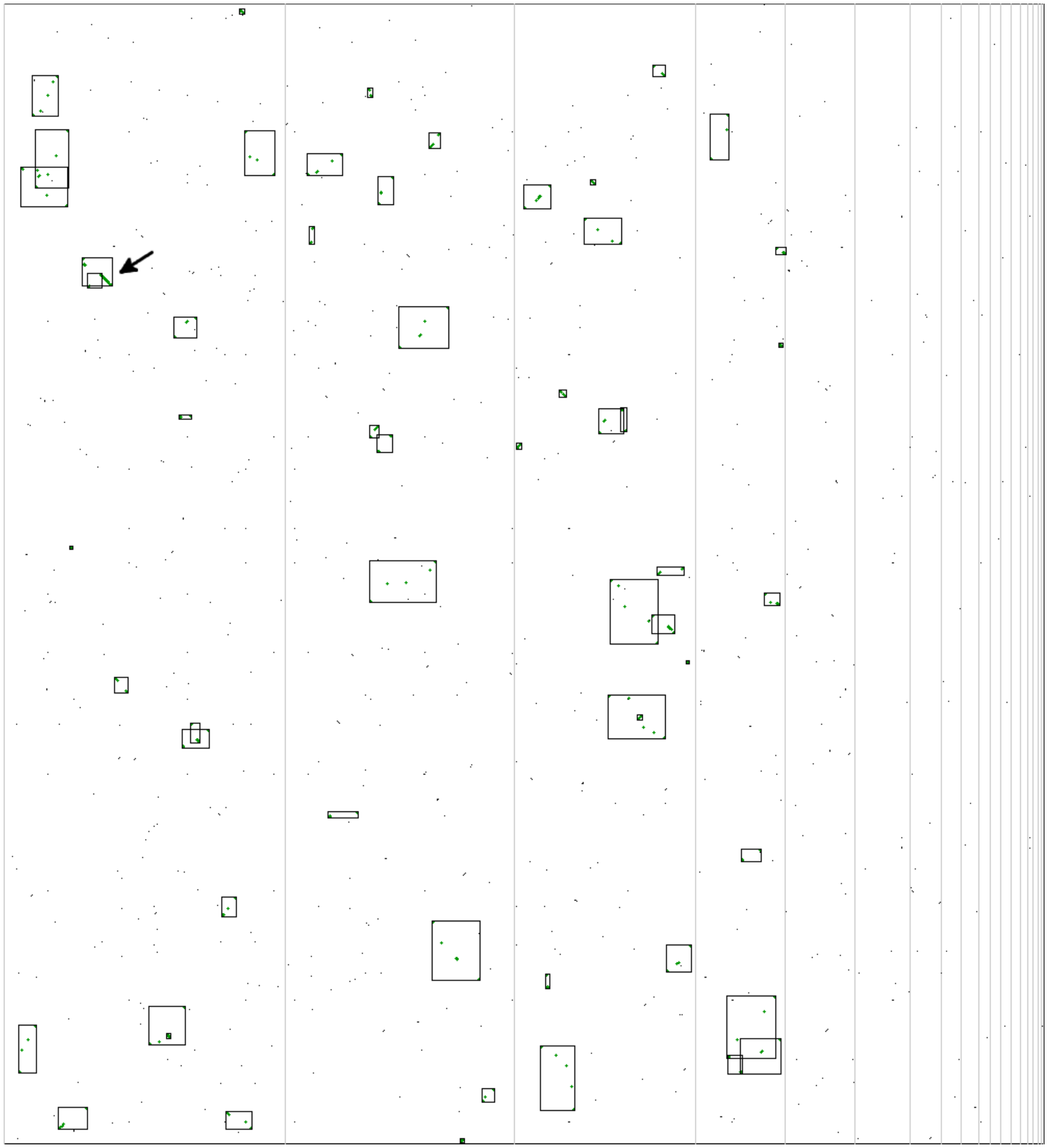

Supplementary Figure S3

Dot-blot of syntenic regions between the genome of the Endosymbiont of *Acanthamoeba* UWC8 and the genome scaffolds of the *Trichoplax* sp. H2 endosymbiont based on gene-models. Green dots surrounded by boxes fulfill criteria of synteny. Black dots represent single pairs that don't fulfill these criteria. The largest syntenic region is a cluster of, mostly, ribosomal protein genes (arrow).

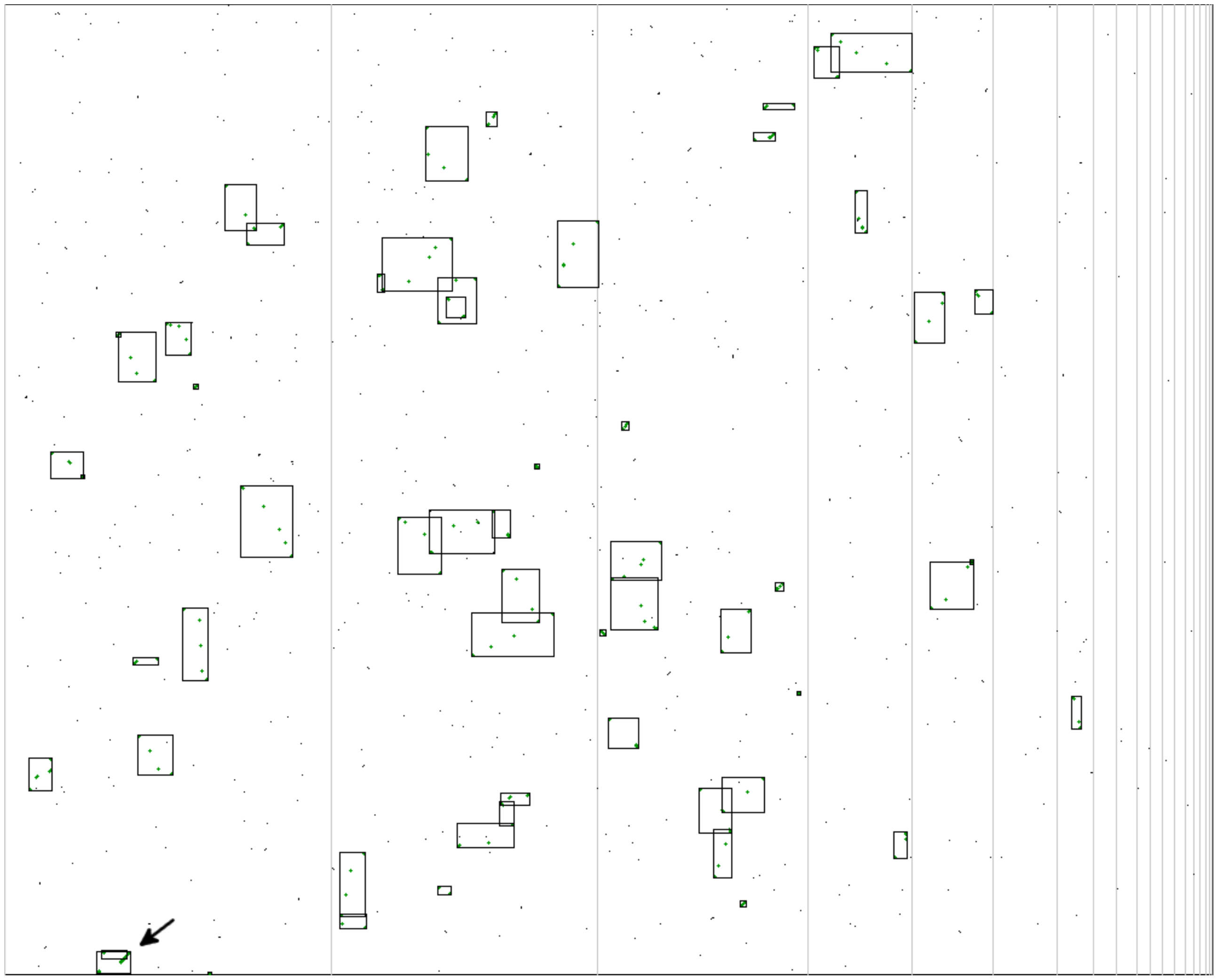

Supplementary Figure S4

Dot-blot of syntenic regions between the genome of *Midichloria mitochondrii* and the genome scaffolds of the *Trichoplax* sp. H2 endosymbiont based on gene-models. Green dots surrounded by boxes fulfill criteria of synteny. Black dots represent single pairs that don't fulfill these criteria. The largest syntenic region is a cluster of, mostly, ribosomal protein genes (arrow).

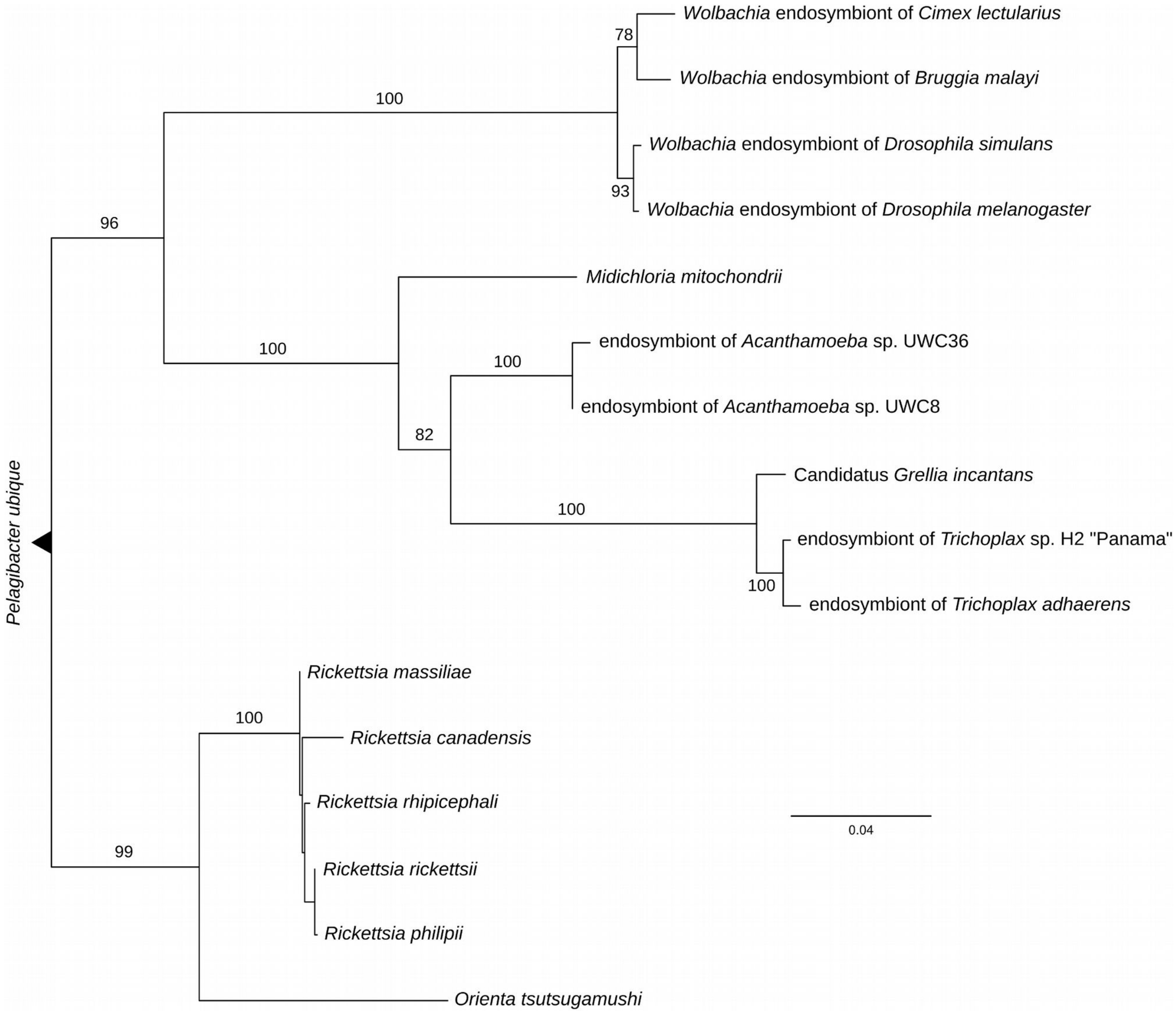

Supplementary Figure S5

16S maximum likelihood phylogenetic tree containing selected taxa of the Rickettsiales, the endosymbionts of *Trichoplax adhaerens* and *Trichoplax* sp. H2 “Panama”, and the recently described placozoan endosymbiont Candidatus *Grellia incantans*. The endosymbionts of *Trichoplax adhaerens* and *Trichoplax* sp. H2 segregate in a clade different from *Grellia incantans*. The tree has been rotted on *Pelagibacter ubique*. Some bootstrap values have been omitted for clarity.

Supplementary References

1. Kamm, K., Osigus, H.-J., Stadler, P. F., DeSalle, R. & Schierwater, B. *Trichoplax* genomes reveal profound admixture and suggest stable wild populations without bisexual reproduction. *Sci. Rep.* **8,** 11168 (2018).
